# An interactive mobile application versus an educational booklet to promote job retention in women undergoing adjuvant chemotherapy for breast cancer: a randomized controlled trial

**DOI:** 10.1186/s13063-022-06580-7

**Published:** 2022-10-03

**Authors:** Victoria S. Blinder, Sujata Patil, Jackie Finik, Della Makower, Monica Muppidi, Wendy G. Lichtenthal, Patricia A. Parker, Maria Claros, Jennifer Suarez, Bharat Narang, Francesca Gany

**Affiliations:** 1 Memorial Sloan Kettering Cancer Center (MSK), New York, USA; 2 Cleveland Clinic, Cleveland, USA; 3 Montefiore Medical Center, New York, USA; 4 Lincoln Medical and Mental Health Center, New York, USA

**Keywords:** Employment, Breast cancer, Disparities, Minority, Income, Cancer survivorship, Financial toxicity, Mobile application

## Abstract

**Background:**

Job loss after a cancer diagnosis can lead to long-term financial toxicity and its attendant adverse clinical consequences, including decreased treatment adherence. Among women undergoing (neo)adjuvant chemotherapy for breast cancer, access to work accommodations (e.g., sick leave) is associated with higher job retention after treatment completion. However, low-income and/or minority women are less likely to have access to work accommodations and, therefore, are at higher risk of job loss. Given the time and transportation barriers that low-income working patients commonly face, it is crucial to develop an intervention that is convenient and easy to use.

**Methods:**

We designed an intervention to promote job retention during and after (neo)adjuvant chemotherapy for breast cancer by improving access to relevant accommodations. Talking to Employers And Medical staff about Work (TEAMWork) is an English/Spanish mobile application (app) that provides (1) suggestions for work accommodations tailored to specific job demands, (2) coaching/strategies for negotiating with an employer, (3) advice for symptom self-management, and (4) tools to improve communication with the medical oncology team. This study is a randomized controlled trial to evaluate the app as a job-retention tool compared to a control condition that provides the app content in an informational paper booklet. The primary outcome of the study is work status after treatment completion. Secondary outcomes include work status 1 and 2 years later, participant self-efficacy to ask an employer for accommodations, receipt of workplace accommodations during and following adjuvant therapy, patient self-efficacy to communicate with the oncology provider, self-reported symptom burden during and following adjuvant therapy, and cancer treatment adherence.

**Discussion:**

This study will assess the use of mobile technology to improve vulnerable breast cancer patients’ ability to communicate with their employers and oncology providers, work during treatment and retain their jobs in the long term, thereby diminishing the potential consequences of job loss, including decreased treatment adherence, debt, and bankruptcy.

**Trial registration:**

ClincalTrials.gov NCT03572374. Registered on 08 June 2018.

**Supplementary Information:**

The online version contains supplementary material available at 10.1186/s13063-022-06580-7.

## Administrative information

Note: the numbers in curly brackets in this protocol refer to SPIRIT checklist item numbers. The order of the items has been modified to group similar items (see http://www.equator-network.org/reporting-guidelines/spirit-2013-statement-defining-standard-protocol-items-for-clinical-trials/).

**Table:** 

Title {1}	An interactive mobile application versus an educational booklet to promote job retention in women undergoing adjuvant chemotherapy for breast cancer: a randomized controlled trial
Trial registration {2a and 2b}.	ClinicalTrials.gov Identifier: NCT03572374
Protocol version {3}	Protocol version 19 approved February 16, 2022
Funding {4}	NIH R37 CA214785 https://reporter.nih.gov/project-details/9448562
Author details {5a}	Victoria Blinder, Memorial Sloan Kettering Cancer Center (MSK) Sujata Patil, Cleveland Clinic Jackie Finik, MSK Wendy Lichtenthal, MSK Patricia Parker, MSK Maria Claros, MSK Jennifer Suarez, MSK Bharat Narang, MSK Della Makower, Montefiore Medical Center Monica Muppidi, Lincoln Medical and Mental Health Center Francesca Gany, MSK
Name and contact information for the trial sponsor {5b}	National Institutes of Health
Role of sponsor {5c}	Sponsor is the principal investigator’s institution.

## Introduction

### Background and rationale {6a}

#### Background

Job loss after a cancer diagnosis can have long-term financial consequences, including difficulty paying bills, accumulation of debt, and bankruptcy [1–4]. Cancer patients who lose their jobs are less likely to adhere to their prescribed treatment, and those who declare bankruptcy have nearly double the risk of death [4].

Breast cancer patients who lack access to adequate sick leave also lack job security. Those who stop working during adjuvant therapy are at increased risk of long-term job loss as their jobs may not be available when they are ready to return to work [5]. Access to sick leave or a flexible work schedule is associated with higher post-treatment job retention [1, 6]. However, low-income minorities and immigrants have limited access to such benefits, leading to higher rates of job loss and financial distress [1, 6]. Therefore, for minority breast cancer patients at risk of job loss, finding a way to continue to work during treatment is critical.

In the Breast Cancer and the Workforce (BCW) study, which focused on immigrant and minority women, we showed that only 57% of pre-diagnosis employed low-income women retained their jobs after treatment completion, compared to 93% of higher-income women [7]. Women who reported that their employer had accommodated their need for treatment were more than twice as likely to retain their jobs [7]. Not surprisingly, low-income women were less than half as likely as higher-income women to report that their employer had accommodated their need for treatment and only one-fourth as likely to retain their jobs [7]. Among those not working at follow-up, treatment and related symptoms were cited as reasons by 84%.

Based on our prior foundational work, we designed TEAMWork (Talking to Employers And Medical staff about Work), an English/Spanish intervention, delivered in the form of a mobile application (app), that supports working during adjuvant breast cancer treatment. Formerly called the BCW Communication App, (https://reporter.nih.gov/search/4GGWW2wtm0iWGqM5zZve9A/project-details/10333219) TEAMWork is designed to educate patients and improve their communication skills with their employers and their oncology providers, thereby increasing access to work accommodations and optimizing symptom control during treatment. By increasing patients’ ability to work during treatment, we believe the app will help them retain their jobs. An app is ideally suited to our study in light of the high prevalence of smartphone use reported by BCW participants (91%) and the barriers our target population faces in accessing support. We propose to test the efficacy of the app as a job-retention tool in a randomized controlled trial comparing the app to an informational booklet.

#### Breast cancer and work

Breast cancer is a common survivable malignancy with the potential for both acute and chronic impact on employment, financial stability, and quality of life. Overall, approximately 70–80% of breast cancer survivors return to work within 3–18 months after diagnosis [8, 9]. Receipt of workplace accommodations is among the strongest predictors of return to work [8, 10]. However, to date, most studies have included primarily US-born, white, middle-income women. Immigrants and minorities who are diagnosed with cancer are less likely to return to work after treatment than are US-born whites. We previously studied a sample of low-income breast cancer survivors and found that Latinas took longer to return to work than non-Latina whites [11]. Although all study participants had been employed pre-diagnosis, fewer than 60% were working 3 years later, a notable contrast to the employment outcomes of wealthier samples.

Differences in job type at least partly explained the ethnic disparity in our study; 24% of Latinas worked as operators/fabricators (e.g., in manufacturing) before diagnosis compared to 5% of non-Latina whites. Survivors in this category were least likely to be working 6, 18, and 36 months after diagnosis. Receipt of chemotherapy was also an important predictor of work outcomes. Women who were still undergoing chemotherapy 6 months after diagnosis were more likely to report that they were not currently working (81% of those receiving chemotherapy vs. 55% of those not in treatment; *p*<0.0001) [5]. Moreover, following the sample for a total of 5 years revealed a concerning long-term trend: 43% of those not working 6 months after diagnosis never returned to work.

#### Disparities in access to work accommodations

Disparities in receipt of workplace accommodations are associated with decreased job retention. Such disparities have been demonstrated with respect to both income and race/ethnicity. We previously showed that low-income women (those with household incomes <200% of the federal poverty level) who were undergoing (neo)adjuvant breast cancer treatment were less likely to have accommodating employers than higher-income women, even after controlling for race/ethnicity, age, and job tenure (OR 0.48, *p*<0.05) [7]. Low-income women were also less likely to retain their jobs 4 months post-treatment (OR 0.25, *p*<0.05), but women with accommodating employers had 2.54 times the odds of retaining their jobs, regardless of income level (*p*<0.05).

Mujahid et al. showed that 24% of Latinas undergoing adjuvant breast cancer treatment experienced job loss, compared to just 10% of blacks and 7% of non-Latina whites (*p*<0.001) [1]. Not surprisingly, access to a flexible work schedule and paid sick leave—both significantly more common among non-Latina whites—were positively correlated with job retention [1]. Receipt of chemotherapy was associated with job loss among Latinas but not non-Latina whites, indicating that work accommodations may diminish the negative impact of chemotherapy on job retention [1]. This research team further identified a disparity on the basis of acculturation among Latinas. Compared to non-Latina whites, Latinas with low acculturation had 10.3 times the odds of stopping work, whereas more acculturated Latinas had only 2.2 times the odds of stopping (*p*<0.005) [6]. Lack of schedule flexibility at work was associated with 19 times the odds of stopping work (*p*<0.001), and Latinas with low acculturation were significantly more likely to lack schedule flexibility than those with high acculturation [6].

Thus, the existing literature has shown that receipt of accommodations is an important predictor of job retention among low-income and minority breast cancer survivors, but access to accommodations varies by income, race/ethnicity, and acculturation. Furthermore, accommodations may be most important for those undergoing chemotherapy.

#### The importance of working during chemotherapy in low-income survivors

In prior research, we showed that low-income breast cancer survivors who undergo chemotherapy are at higher risk of not working for 5 years after diagnosis than are women who do not undergo chemotherapy [5]. This difference in outcome is most likely due to taking time off during treatment in the absence of sick leave benefits. Nationwide, only 21% of low-wage workers have access to paid sick leave [12]. Women without sick leave who stop working have no guarantee that their jobs will be available once they are ready to return, and as a result, they are at risk of permanent job loss. The problem is exacerbated for workers in the informal sector (i.e., those who work “off the books”). Workers in this situation have no protections in case of illness under the Americans with Disabilities Act or the Family and Medical Leave Act [13–15]. In this setting, increasing their ability to work during chemotherapy could shield them from the potentially catastrophic consequences of long-term job loss. However, patients with a higher treatment-related symptom burden have lower self-reported work ability [16]. Therefore, improving symptom control during treatment is vital to optimizing patients’ ability to work.

#### Consequences of job loss in cancer survivors

Breast cancer survivors’ job loss has been positively correlated with short- and long-term financial distress [1, 10]. We previously showed that, for low-income survivors, not working was associated with financial distress for 5 years after diagnosis [17]. Banegas et al. showed that 3% of cancer survivors aged 18-64 reported filing for bankruptcy, and 34% reported that they or a family member had gone into debt because of cancer [2]. The risk of bankruptcy or debt was highest among survivors who were young, uninsured, or unemployed. Similarly, Ramsey et al. analyzed 1995–2009 bankruptcy filings in Washington State and found that 2.2% of people filed for bankruptcy protection after being diagnosed with cancer, a rate 2.65 times higher than in matched non-cancer controls [3]. Cancer patients who were younger, female, or non-white were at the highest risk of bankruptcy.

The impact of financial distress on health outcomes cannot be overstated. In a subsequent analysis of the Washington State data, Ramsey et al. showed that cancer patients who filed for bankruptcy had 1.79 times the risk of death compared to those who did not file for bankruptcy [4]. The analyses were unchanged when patients with distant metastases at diagnosis were excluded. Other investigators have shown that financial toxicity of treatment and job loss are linked to decreased adherence, such that this increased mortality is likely, at least in part, a consequence of financial limitations, including bankruptcy [18–21].

#### Self-efficacy and communication skills in interactions with a physician and/or an employer

Higher perceived efficacy in patient-physician interactions is associated with improved resolution of treatment-related symptoms (e.g., nausea) and increased treatment adherence [22, 23]. Moreover, symptom burden is inversely associated with work ability and post-treatment job retention [16, 24]. We propose that communication behaviors can be shaped through interventions using components of social cognitive theory, a widely accepted theory of health behavior change that focuses on developing patients’ sense of self-efficacy. Social cognitive theory highlights the connection between environmental factors (e.g., social and physical situations), a patient’s personal factors (e.g., thoughts and feelings), and their health behaviors. Our theory-driven mobile app uses well-established intervention components grounded in social cognitive theory, including skills training, observational learning, and reinforcement, to increase patients’ self-efficacy in communicating with their employers and healthcare providers [25, 26].

For many women undergoing breast cancer treatment, working during chemotherapy is a realistic goal, if they can access work accommodations and optimize symptom control. To achieve this, the survivor must be able to communicate effectively with her employer and with the oncology team. Higher self-efficacy in interacting with a physician could help women access resources such as better antiemetics, if needed. Similarly, higher self-efficacy in interacting with an employer could help women negotiate for accommodations (e.g., a nanny could arrange part-time coverage and keep working during treatment).

Several programs have successfully taught patients to communicate more effectively with providers [27, 28]. Approaches include coaching, communication strategy descriptions, and prompt sheets [29–33]. A systematic review found that patient-targeted interventions enhance patient participation in oncology consultations [32]. These approaches are effective regardless of English proficiency, as demonstrated by an English/Spanish patient-targeted intervention which led to improved patient-physician communication and increased physician implementation of patients’ survivorship needs [34]. Similar research with respect to the workplace setting is lacking. In a small qualitative study, a DVD was designed to improve cancer patients’ access to work accommodations using patient-physician communication strategies adapted for the work setting [35]. Participants reacted favorably to the DVD, but employment outcomes were not assessed. Moreover, a DVD is not ideal for our target population due to its limited amenability to individual tailoring based on job type and other characteristics, and because patients lack free time to watch. In contrast, a mobile app is convenient (it may be used anywhere and for short bursts of time) and allows users to tailor the intervention to their individual needs.

#### Use of mobile technology to deliver the intervention

Given the time and transportation barriers that low-income working patients commonly face, it is crucial to develop an intervention that is convenient and easy to use. Smartphone use is common, and app-based interventions are feasible in minority and/or low-income urban populations [36, 37]. Moreover, black and Latino cell-phone owners are more likely than non-Latino whites to access the Internet via their phones [38]. Among BCW participants, 91% had smart phones, 89% used the Internet, and 82% used the Internet daily.

#### Conceptual framework and study rationale

The conceptual framework for work outcomes in cancer survivors developed by Wilson and Cleary and adapted by Steiner is useful in describing the relationships between the sets of variables that affect job retention [39, 40]. This model includes two sets of modifying characteristics. “Characteristics of the individual” include sociodemographic characteristics, comorbid conditions, and personal goals/values. “Characteristics of the environment” include work accommodations and social support. Our conceptual framework combines Steiner’s description of work outcomes with Bandura’s model of social cognitive theory, resulting in an intervention to shape communication behavior and ultimately improve work outcomes.

Low-income immigrant and minority breast cancer survivors face the dual challenges of coping with the toll of cancer and loss of employment because of their treatment needs. Without support, those who need to maintain employment struggle to do so, resulting in higher rates of job loss and financial and psychological distress, potentially with long-term or even permanent consequences [1, 6, 41, 42]. Through this project, we will study an innovative, evidence-based, culturally-sensitive, and relevant intervention to support the maintenance of employment.

### Objectives {7}

The specific aims of this study are:

- Aim 1: To refine the mobile app through focus groups (to vet app content) and usability testing (to optimize app functionality) in English- and Spanish-speaking women. Focus groups will be conducted with women who were employed prior to diagnosis or at the time of consent to the BCW study [7], and who completed (neo)adjuvant chemotherapy for breast cancer. Usability testing will be conducted in individuals who were employed prior to diagnosis and are undergoing or plan to undergo treatment for a diagnosis of cancer.
- Aim 2: To evaluate the effect of the mobile app on job retention after completion of adjuvant therapy for breast cancer among English- and Spanish-speaking women who were employed prior to diagnosis and plan to undergo chemotherapy.
  - *Hypothesis:* Participants randomized to the app will have higher rates of employment four months, 1 year, and 2 years after treatment completion compared to those randomized to receive the booklet.
- Subaim 1: To evaluate whether the effect of the app on job retention varies by characteristics including chemotherapy regimen, symptom burden, race/ethnicity, language, and job type.
- Aim 3: To evaluate the effect of the mobile app on confidence in asking an employer for accommodations and on receipt of workplace accommodations during and following (neo)adjuvant therapy.
  - *Hypothesis:* Participants randomized to the app will have greater improvements in confidence in asking an employer for accommodations and will be more likely to receive requested workplace accommodations compared to those randomized to receive the booklet.
- Aim 4: To evaluate the effect of the mobile app on patient efficacy in communicating with the oncology provider and self-reported symptom burden during and following adjuvant therapy.
  - *Hypothesis:* Participants randomized to the app will have higher efficacy in communicating with the provider and lower self-reported symptom burden based on the NCI Patient Reported Outcomes Common Terminology Criteria for Adverse Events (PRO-CTCAE®) system compared to those randomized to receive the booklet.
- Exploratory aim 1: To evaluate the effect of the mobile app on cancer treatment adherence.

### Trial design {8}

Prior to enrolling participants in the randomized controlled trial (RCT), we will refine (1) the usability details of the app and (2) the content of the app, based on focus group feedback, to optimize ease of use in newly diagnosed English- and Spanish-speaking patients matching the eligibility criteria that will be applied in the RCT. To ensure that the app is understandable, relevant, and suitable for use prior to and during chemotherapy, this first part of the study will include participants who have not yet started chemotherapy as well as those who have undergone one or more cycles of treatment. The app and the information booklet will be modified in an iterative fashion between waves of usability testing and focus groups. Because certain aspects of the app may change based on the results of usability testing, participants in the first part of the study will not be followed beyond the usability testing and completion of the Early User Experience Survey, and they will not be included in analyses of study outcomes.

To optimize the diversity of usability testers across education level, job type, and Spanish regional variation, usability testing, which focuses on the technological characteristics of the app, was expanded to include community members who did not have a cancer diagnosis.

In the second part of the study, we will evaluate the effect of the TEAMWork app on the employment status of breast cancer survivors in the short term (4 months after completion of adjuvant treatment) and in the long term (1 and 2 years after completion of treatment). Participants will be randomized 1:1 to receive the TEAMWork app or an informational booklet (control) that includes all the components of the intervention that can practicably be delivered in print form. TEAMWork is a two-pronged intervention; it includes two “menus,” one of which focuses on interactions with the employer and the other with the oncology providers. Additional details regarding the intervention are included below.

## Methods: participants, interventions, and outcomes

### Study setting {9}

Participants will be recruited at Memorial Sloan Kettering Cancer Center (MSK, an NIH-designated cancer center with locations in New York City and surrounding suburbs), NYC Health & Hospitals/Lincoln Medical Center (a county hospital), and Montefiore Medical Center (a large academic medical center located in the Bronx that serves an ethnically and socioeconomically diverse population). We will recruit potential participants from oncology clinics (medical and surgical), patient meetings, and workshops of participating hospitals and practices.

### Eligibility criteria {10}

Eligibility criteria for each part of the study are summarized below.

Patient usability testing inclusion criteria:

- History of a cancer diagnosis
- At least 18 years of age
- Male or female
- The ability to give informed consent in English or Spanish
- Able to use and read a smartphone (iPhone or Android)
- Has a smartphone (iPhone or Android)

Community usability testing inclusion criteria:

- At least 18 years of age
- Male or female
- The ability to give informed consent in English or Spanish
- Able to use and read a smartphone (iPhone or Android)
- Has a smartphone (iPhone or Android)

Focus group and interview inclusion criteria:

- Cohort 1
  - Completed chemotherapy treatment for stage I–III breast cancer
  - Age 18 to 64 (inclusive) at time of diagnosis
  - Female
  - Paid employment (full time or part time) in the three months prior to diagnosis or at time of consent to the BCW study [7]
  - The ability to give informed consent in English or Spanish
  - Able to use and read a smartphone (iPhone or Android)
  - Has a smartphone (iPhone or Android)
- Additional inclusion criteria for cohort 2
  - Participant was covered by Emergency Medicaid at the time of diagnosis and/or at any point during their treatment for breast cancer.

RCT inclusion criteria:

- Localized invasive breast cancer (not stage 0 or stage IV)
- Planning to undergo or undergoing adjuvant or neoadjuvant chemotherapy
- Age 18 to 64 (inclusive)
- Female
- Paid employment (full time or part time) at time of consent
- The ability to give informed consent in English or Spanish
- Able to use and read a smartphone or tablet (e.g., iPad, iPhone, or Android)
- Has access to a smartphone or tablet (e.g., iPad, iPhone, or Android)
- RCT exclusion criteria:
- Distant recurrence (metastasis) of breast cancer

### Who will take informed consent? {26a}

Potential participants will be identified by a member of the patient’s treatment team, the protocol investigator, or the research team. The principal investigator and study team may also screen the medical records of patients with whom they do not have a treatment relationship for the limited purpose of identifying patients who would be eligible to enroll in the study and to record appropriate contact information to approach these patients regarding the possibility of enrolling in the study (see request for limited waiver of authorization below). A trained member of the research team who is a consenting professional, generally a clinical research coordinator (CRC), will be responsible for screening and consenting participants.

### Additional consent provisions for collection and use of participant data and biological specimens {26b}

As described in the study consent documents (including the verbal consent), deidentified participant data may be used in ancillary studies (i.e., for research that has not been described explicitly to the participant), and they may be shared with other investigators for future research.

## Interventions

### Explanation for the choice of comparators {6b}

#### Information booklet (control)

Participants randomized to the control arm will receive a booklet that includes the information available in the app that can practicably be converted to paper. These participants will not have access to the multimedia aspects of the intervention, such as the videos, but they will have all of the relevant information available in the app described below, including suggestions for accommodations, written templates for letters, links to websites, information about legal protections, and contact information for pro bono legal assistance. The booklet will also contain information about chemotherapy, radiation therapy and surgery, recommendations for management of common symptoms, and advice for communicating with the clinic team. The information booklet will be provided entirely on paper, although participants may independently access websites recommended in the booklet. The booklet content will mirror the app with regard to cultural responsiveness and appropriateness for different job types and characteristics.

### Intervention description {11a}

Our intervention was developed based on data provided by BCW study [7] participants with input from content experts, including our study advisory panel and co-investigators. The 2-pronged approach of the intervention is operationalized through two menus, one focused on interactions with the employer and the other with the clinic team. Each menu has a list of features from which participants can choose to learn about a particular topic. A “My notes” button allows participants to take notes directly on the app. These notes will not be available to the research team, such that participants may use the tool without concerns about privacy.

The work section of the “Learn” tab includes sample videos using trained actors to demonstrate how to approach an employer to request accommodations. Additional features include suggestions for accommodations that may be helpful, templates for letters participants can use when requesting accommodations, links to relevant websites, information about legal protections, and contact information for lawyers and firms that provide pro bono assistance.

The breast cancer section of the “Learn” tab includes information about different common toxicities associated with chemotherapy as well as some information about “what to expect” from radiation therapy and surgery (participants undergoing neoadjuvant chemotherapy will not yet have undergone surgery), recommendations for the management of some of these symptoms (participants reporting symptoms considered urgent will be instructed to contact their oncology providers right away), and advice for communicating with the clinic team (e.g., what kind of information about symptoms the patient should record to share with the provider, including frequency, severity, and associated symptoms and circumstances). This menu also includes examples of ways in which the oncology provider can be helpful with respect to the participant’s work (e.g., providing documentation about the treatment plan or requesting specific accommodations on behalf of the patient) and templates for letters from the oncology provider to the employer. Videos in this part of the app will show examples of interactions with oncology providers, including patients asking for work-related advice and documentation from the provider. The app will also include advice about how to navigate language barriers, including information about accessing an interpreter in the clinic. The app will also show participants the “Tip of the Day,” which will provide helpful information to the TEAMWork app user.

#### Cultural tailoring of the app

The content of the app was developed with the input of both Latina and non-Latina BCW study [7] participants. The text will continue to be adapted and translated using language that is neutral across Latino groups and lacking in idioms, such that people will relate to the content independent of country (and culture) of origin. The Spanish language videos will include actors of different national origins spanning the Latin American countries and territories from which the greatest numbers of Latinas immigrated to New York City. The scripts and expressions used will be familiar to all Spanish-language users but with clear regional derivation. During the usability testing, we will include English and Spanish speakers of different cultural and national backgrounds to ensure that the content is understandable and relatable to our diverse target population.

#### Appropriateness of the app for different job types

The app includes information that is relevant to women in a variety of job situations, many of which are common to different job types, based on a sample from the BCW study [7]. Rather than being sorted by job type (e.g., “retail clerk”), the information is sorted by job characteristics (e.g., “My job requires me to be on my feet for long periods of time”). By sorting the information in this way, participants will more easily be able to access information that is relevant to their specific situation. For example, many retail clerks stand for long periods of time at work. However, this job characteristic is far from universal across all settings in which a retail clerk might work. On the other hand, a nanny or day-care worker who cares for newborn babies may not need to stand for long periods at a time, whereas one who cares for toddlers may need to do so. The app includes a variety of different job characteristics relevant to our target population.

### Criteria for discontinuing or modifying allocated interventions {11b}

Any patient who experiences significant study-related distress or asks to discontinue participation will be removed from the study.

### Strategies to improve adherence to interventions {11c}

Usage tracking analytics will be provided to the study team. These include whether a user accessed the app, which sections were accessed most/least, and the duration of access. During the first 8 months after their study enrollment, participants who have not accessed the app at all during the preceding week will receive targeted “non-use” push notifications encouraging them to use the app and highlighting various app features. Participants may choose to opt out of these notifications. After 8 months, participants may continue to use the app, but they will no longer receive “non-use” notifications, as most will have completed chemotherapy by then.

### Relevant concomitant care permitted or prohibited during the trial {11d}

A potential limitation is the possibility that participants enrolled in the study will access resources extrinsic to the study, which might also affect employment outcomes and symptom control. However, if successful, our intervention will be implemented in a “real-world” setting, in the context of other such resources. Randomization of study participants will help minimize potential bias resulting from the contamination of our study design by external resources. In addition, we will ask participants about other resources they may have accessed and incorporate these data into sensitivity analyses to see how they may have affected our results. This information will be included when we report our study findings to ensure transparency of our study procedures and results.

### Provisions for post-trial care {30}

All research staff will be trained to identify signs of psychological discomfort in the participants. Any patient reporting significant distress (or indicating distress through other signs) will be referred to a licensed or board-certified mental health provider for additional evaluation, support, and/or referrals as needed.

### Outcomes {12}

The primary endpoint of the trial is self-reported employment status 4 months following the completion of treatment. This is the same primary endpoint as was used in the BCW study [7]; timing the assessment of employment status based on the end of treatment rather than the date of diagnosis provides an assessment that is comparable across participants, regardless of the timing between diagnosis and start of treatment. Depending on disease characteristics, provider recommendations, and patient preferences, participants will undergo different chemotherapy regimens with differences in cycle length and total number of cycles. They, therefore, will vary in their overall chemotherapy treatment period. Our endpoint of employment status 4 months following the completion of all treatment, consequently, has a shared interpretation across types of therapy. Two additional assessments will allow us to measure long-term employment status (1 and 2 years after completion of treatment). Patients working full-time or part-time will be considered to be working (job retained) regardless of whether they worked full-time or part-time at baseline.

Secondary study endpoints include change in self-efficacy to ask an employer for accommodations, receipt of workplace accommodations, change in perceived efficacy in patient-physician interactions (PEPPI), and symptom burden. We will ask participants about accommodations requested at baseline and at each on-treatment and post-treatment survey. Those who report asking for accommodations will also be asked to report on whether their request was granted, including whether the request was granted in full, in part, or if they were given a different accommodation instead. Symptom burden will be assessed using relevant items from the Patient-Reported Outcomes version of the Common Terminology Criteria for Adverse Events (PRO-CTCAE®). Details of these analyses are included below. These four secondary outcomes will be evaluated with respect to their relationship with the primary outcome.

The endpoint for the exploratory aim is treatment adherence based on relative dose intensity (RDI), a measure that incorporates both chemotherapy dose-reductions and treatment delays, and for which a standard exists that can be compared across chemotherapy regimens. RDI is expressed as a percent; the commonly accepted threshold below which adjuvant chemotherapy for breast cancer is less effective is 85% [43–45].

### Participant timeline {13}

Timing of procedures and measures for the RCT are provided in Table 1. On-treatment surveys will be administered 5–10 weeks after starting chemotherapy, which will allow for an “on-treatment” assessment that accounts for the wide variation in duration of standard adjuvant chemotherapy regimens for breast cancer. Additional details on measures are included in the section titled “Plans for assessment and collection of outcomes {18a}” below.

**Table 1:**
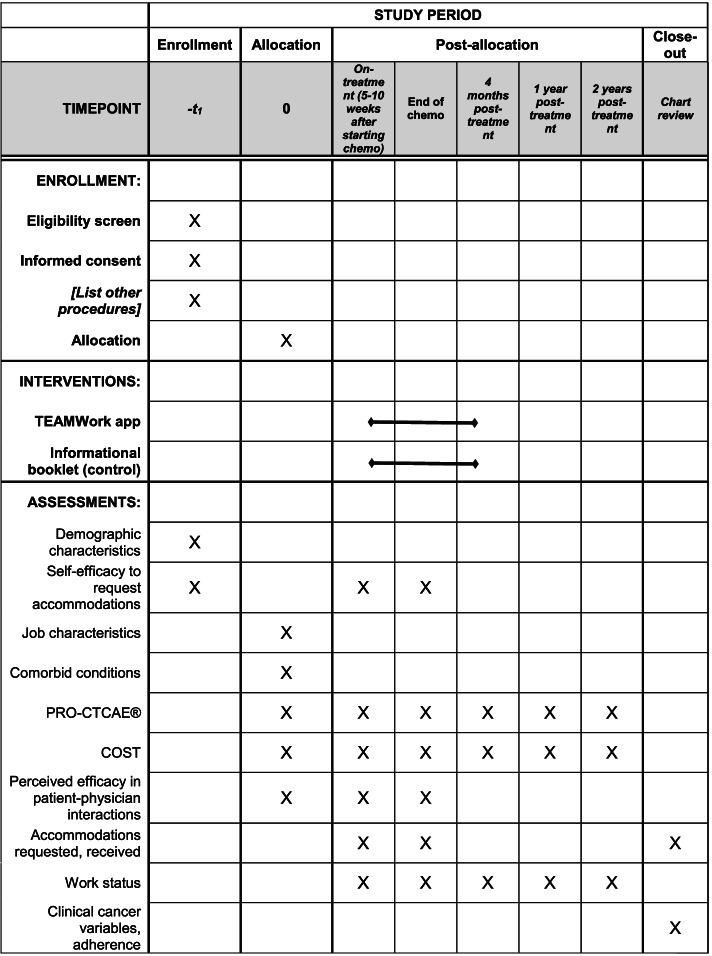
SPIRIT figure for randomized controlled trial

### Sample size {14}

*Sample size for Aim 1:* Based on the standards for usability testing codified by Jakob Nielsen and recommended by the Department of Health and Human Services [46–48], an iterative design using 3 waves of 3–4 participants each is needed to optimize usability when 2 distinct groups of users are tested (i.e., English and Spanish speakers). Therefore, we anticipate that we will complete usability testing with 18-24 participants. We will do additional usability testing if significant usability issues continue to be identified during the third wave of usability testing.

*Sample size for Aim 2:* The sample size calculations for the proposed study are based on the findings of the BCW study [7], our prospective, longitudinal, observational study of disparities in employment outcomes. The study sample in BCW [7] is similar to that anticipated for the proposed study (based on eligibility criteria and recruitment sites). In BCW [7], 82% of participants completed 4-month follow-up surveys, 79% completed 1-year follow-up surveys, and 58% completed 2-year follow-up surveys. Based on these data, we anticipate that at least 80–85% of participants in the proposed study who complete baseline assessments will also complete the 4-month assessment and, therefore, be evaluable for the primary endpoint. In contrast to the BCW study [7], which was observational only and in which there was no contact with participants between the baseline and 4-month post-treatment survey, the proposed study is testing an intervention and will include several points of contact throughout the treatment period. We anticipate greater participant engagement in the proposed study and, therefore, higher retention than observed in the BCW study [7]. Nevertheless, for the purposes of sample size calculation, we will use the BCW study [7] retention statistics as a conservative estimate. For the 1- and 2-year endpoints, we expect retention to be at least 80% and 60%, respectively.

In BCW [7], approximately 50% of pre-diagnosis employed women who underwent chemotherapy were working 4 months after treatment completion. The difference in outcome between participants who did and did not have access to paid sick leave through their jobs was approximately 19% and was statistically significant (*p*=0.01). The mechanism of action of our intervention is hypothesized to be that working during the treatment period will provide job security to women and, therefore, lead to long-term job retention. Because paid sick leave provides similar job security, we will use a conservative effect size of 15% for our proposed intervention.

To identify an improvement of 15% in job retention 4 months post-treatment (i.e., 65% job-retention) in the arm randomized to receive the app, we would require 167 evaluable participants in each arm based on a 2-sided test with a 5% type I error and 80% power. We plan to recruit 420 women to obtain primary outcome data from 334 participants (assuming >80% retention 4 months post-treatment). *As noted above, we have estimated 80% retention 1 year post-treatment; we anticipate having higher retention 4 months post-treatment. Thus, we will have at least 80% power and 5% type I error to detect improvements in job-retention of 15% or more 1 year post-treatment. We expect further attrition by year 2. With a conservative 60% retention rate at 2 years, we will have 80% power to detect a difference of at least 17% for job retention at year 2. Using mixed models and important covariates, the detectable effect size for this comparison will likely decrease.*

*Sample size for sub-aim 1:* Characteristics of interest include chemotherapy regimen, symptom burden, race/ethnicity, language, and job tasks. We anticipate that, for example, 35% of participants in the study will have jobs that involve standing for long periods of time. This distribution will allow us to determine whether the association between the app and job retention varies by this job task.

*Sample size for Aim 3:* The percent of participants who express confidence in asking an employer for accommodations will be compared by study arm. This outcome will be evaluated at the mid-point and on the last day of chemotherapy. As a conservative estimate, we expect that 80% of participants will have evaluable data at each of these time-points. Confidence in asking an employer for accommodations based on participant answers to a 5-item scale (see Data collection and management below) will be dichotomized based on the cutoff established in the observational BCW study [7]. Thirty percent of BCW study [7] participants improved their confidence in asking for accommodations between baseline and 4 months post-treatment. In the current study, with a sample size of 167 per arm, we will have 80% power to identify an improvement in confidence of 15% (e.g., 30% of control-arm participants and 45% of those who receive the app) based on a 2-sided test with a 5% type I error.

*Sample size for Aim 4:* Perceived efficacy in patient-physician interactions (PEPPI) at baseline, mid-treatment, and end of chemotherapy treatment will be compared by study arm in 3 *t*-tests. The PEPPI is a 5-item scale (with range 0–50); higher scores indicate greater self-efficacy. In a similar patient sample, the mean was 38 (standard deviation=12). Based on the retention projections described above, we expect to have data for at least *n*=167 per arm at each of the 3 time-points. For each analysis, we will be able to detect a difference from 38 to 42 (>85% power, 2-sided type I error of 5%).

### Recruitment {15}

Participants in all phases of the study (focus groups, interviews, and RCT) may be consented verbally over the phone or in person. All patient recruitment will be done by research staff who are consenting professionals with (when necessary) a volunteer affiliation with the collaborating study site. Bilingual research staff (Spanish-English) who are consenting professionals will recruit Spanish-speaking patients.

Data collected from patients who are ineligible will be maintained linked to an anonymized ID for the purposes of clinical trials reporting as per the Consolidated Standards of Reporting Trials (CONSORT) guidelines to optimize rigor and transparency in study results [49]. Patients who meet the criteria for inclusion will proceed with the informed consent process and, if they choose to participate, will be enrolled in the study.

A signed written informed consent form is not required for this study, based on the Code of Federal Regulations Title 45, Part 46, Subpart A, which states that an IRB may waive the requirement for an investigator to obtain a signed consent form for some or all subjects if it finds that the research presents no more than minimal risk of harm to subjects and involves no procedures for which written consent is normally required outside of the research context. Therefore, to help reduce subject burden and more efficiently use research resources, consents obtained for this study will be verbal. In all cases, participants will be given a written document (study information sheet) outlining their commitment and study procedures.

#### Usability testing participants

Usability testing participants may be recruited through oncology clinics at MSK, NYC Health & Hospitals/Lincoln Medical Center, and Montefiore Medical Center. Additionally, usability testing participants may also be recruited by the study team through the Memorial Sloan Kettering Cancer Center food pantries.

#### Community usability testing participants

In addition to the usability testing recruitment described above, our recruitment strategy for Spanish community usability testing participants will focus on the recruitment of a community sample of native Spanish speakers of different national backgrounds with special attention to occupational groups that are categorized as service and production type jobs (e.g., nanny, nail technicians/manicurists, and restaurant workers) as determined by our aggregation of the Bureau of Labor Statistics Standard Occupational Classification Policy Committee (SOCPC) [50]. The Immigrant Health and Cancer Disparities Service (IHCD) at Memorial Sloan Kettering Cancer Center, in which our research team is housed, has a longstanding relationship with the New York City Spanish-speaking community and community partners, to facilitate access to health services through its ongoing health fairs and education activities. Several of these organizations serve as regular meeting points for the Spanish-speaking community. We will utilize these existing community partnerships (e.g., the Mexican Consulate) to arrange a date and time for study personnel to approach and potentially enroll employees and/or community members on site. In addition, we will work with informal networks (e.g., groups of nannies, nail-salon workers) to advertise the study and set up a time to meet with interested participants. Whenever possible, usability testing will be done on site at MSK. We recognize that this location may be inconvenient for some participants, however, and if necessary, we will meet participants in appropriate public spaces (e.g., a café) that is near to the participant’s workplace.

Because medical information will not be collected about these participants, the study team sought and was granted a waiver to obtain informed consent from participants in the community-based usability testing phase of the study, based on federal regulations 45 CFR 46.116(d) and 21 CFR10.115(g) [2].

#### Focus group and interview participants

Participants in focus groups and interviews may include, in addition to patients treated at the clinical sites described above, patients identified through the community through the Integrated Cancer Care Access Network (ICCAN) or through breast cancer support organizations, such as LatinaSHARE, and patients who were previously enrolled in the BCW study [7]. Patients who meet the criteria for inclusion will be told about the study and, if they choose to participate, will be scheduled to attend a focus group or for an interview (in-person or over the phone) and sent a copy of the TEAMWork booklet by email and/or mail for their review prior to the focus group or interview. At the start of the focus group or interview, patients will be informed of the details of the protocol, including study overview and aims, study procedures, and the risks involved. After this open discussion, each participant will be consented individually by a consenting professional, in person or via phone.

#### RCT participants

The research team will contact the treating physician (surgeon or medical oncologist, depending on who has most recently seen the patient in clinic) to verify that it is acceptable for the team to approach each patient about the study. If at the time of the initial medical oncology consultation no definitive decision is made regarding whether a potential participant will undergo chemotherapy, the research staff will communicate with the oncology provider regarding the final recommendation and patient decision regarding systemic therapy.

Research staff will approach participants at a post-operative visit with the surgeon, at their initial consultation with the medical oncologist, or a patient meeting or workshop (after obtaining permission from the meeting or workshop leader) to describe the study. Staff may also call patients about the study, and leave a voicemail, when applicable.

Our target study population is often hard to reach due to changes in phone number, loss of phone service, limited phone service, treatment side effects, lack of fixed working schedules, and change in address. In prior research, our team has experienced difficulties contacting members of our target population for an initial approach. However, after multiple attempts, we generally have success reaching our population. As a result, potential participants may be contacted at least 3 times. If the team is unable to contact the patient by phone, the team may approach the patient in clinic and offer the opportunity to learn more about the study. If the patient declines to participate by phone, no further attempts will be made to contact the patient.

If an individual is interested in the study, the research staff will assess their eligibility by going through the inclusion and exclusion criteria and asking the individual a set of screening questions. Answers will be recorded in the screening log and/or on the participants’ eligibility checklist. Patients approached who do not meet the eligibility criteria or decline participation will be asked about their self-efficacy to request accommodations at work and their answers will be recorded.

After verifying eligibility and completing the informed consent process with those who wish to participate, study staff will give participants instructions on how to log onto the study website to complete an online survey at a later date, directions on how to complete the survey on an iPad in clinic, or administer the survey interview-style. At the time of enrollment, we also will ask participants to provide contact information for themselves as well as for two emergency contacts who we may reach in case we are unable to contact the participant for administration follow-up surveys. We will call each participant who has not completed a baseline survey within 1 week of enrollment to offer the opportunity to complete a survey interview-style (if participant prefers that to an online survey) or to remind the participant to log onto the website. If the participant prefers to complete a survey interview-style, the research staff will administer the survey and enter participant responses directly into the online survey in real time, on the participant’s behalf.

## Assignment of interventions: allocation

### Sequence generation {16a}

Study participants will be assigned to a study arm via a randomly generated list on REDCap using the Clinical Research Database (CRDB) system at Memorial Sloan Kettering Cancer Center, which allows for off-site and off-hours randomization, as needed. Randomization will be stratified by language (English and Spanish) and recruitment site. The recruitment sites for this study are MSK, Lincoln Medical and Mental Health Center, and Montefiore Medical Center.

### Concealment mechanism {16b}

Study participants will be asked to complete their baseline survey prior to being informed of their study arm assignment.

### Implementation {16c}

Once the participant is consented to the study, the consenting professional will sequentially assign the participant to the intervention or the control arm identified in the randomization list, provided by the Department of Epidemiology and Biostatistics at MSK using a computer program to generate the list of randomization assignments in REDCap.

## Assignment of interventions: blinding

### Who will be blinded {17a}

Due to the nature of this study, it is not possible to blind participants or providers to the assigned treatment. Any tainting of our findings due to non-blinding would be anticipated to diminish our finding of a difference between treatment arms, however, rather than supporting a finding of a difference where there is none. All study procedures will be conducted to ensure complete transparency at the conclusion of the study, such that a Consolidated Standards of Reporting Trials (CONSORT) diagram may be constructed to describe recruitment and randomization in detail [49]. Moreover, the procedures followed, including the details of the interventions, will be described in rigorous detail and relevant materials (e.g., the app and information booklet) will be made available so that others may reproduce and extend our findings.

### Procedure for unblinding if needed {17b}

Participants will not be blinded regarding their assigned intervention arm.

## Data collection and management

### Plans for assessment and collection of outcomes {18a}

#### Usability testing

Clinical research coordinators (CRCs) will record the participant’s verbal and non-verbal expressions while performing several tasks using the TEAMWork App. Participants will be permitted to use the TEAMWork Help Section as a guide when having difficulty completing a task. Participants will be asked to complete an Early User Experience Survey after the usability test. The survey, which will take approximately 10–15 min to complete, asks about their experiences using the app, including which features they like or dislike and if any aspects of the app’s content or functionality are unclear or difficult to use. Participants will also be asked if there is anything they would change, remove, or add. This survey also includes questions about participant demographics and their cancer history. This survey will be self-administered electronically on a study iPad. Participants not able to complete this survey in clinic will be given a link they can use to take the survey from home within 1 week of their consent date. In total, the usability testing procedures and Early User Experience Survey will take approximately 30–60 min to complete. Due to time constraints in clinic and the ongoing adjustment of the TEAMWork app during this phase, not all tasks and questions will be completed by each usability testing participant.

#### Focus groups/interviews

##### Focus groups

Introductions and a set of guidelines will be presented to participants at the beginning of the focus groups. Once all participants have acknowledged this set of rules, the facilitator will begin with questions from each section. A focus-group guide will be used as a blueprint for key discussion topics. Due to time constraints, it is possible that not all questions will be addressed. The moderator will guide the groups’ conversation to stay on time while providing information on all the topics, as listed below.

1. Content of the mobile app (20 min)
2. Content of the videos (50 min)

Each focus group will last approximately 1.5 h, including the introduction and guidelines (10 min), topics (70 min), and wrap-up (10 min).

Audio recordings of focus group discussions will be transcribed (and translated, if appropriate) for subsequent qualitative analysis.

##### Interviews

Interviews will use the same guide as the focus groups as a blueprint for key discussion topics. Due to time constraints, it is possible that not all questions will be addressed. The interviewer will ask the participant their thoughts on the content of the mobile app and content of the videos. Each interview will last approximately 1 h and 30 min. Audio recordings of the interviews will be transcribed (and translated, if appropriate) for subsequent qualitative analysis.

#### RCT surveys and measures

Baseline and on-treatment surveys will be administered by study staff over the telephone or in person (staff will enter participant-reported data into the study electronic database in real time), self-administered by patients in clinic using dedicated study iPads, or self-administered at home, based on participant preference. A summary of the timing of surveys for the RCT is included in Table 1, and the surveys are further described in detail below. Participants will be encouraged to complete the surveys in the clinic, but those unable to do so will be provided with a link they can use to access a survey from home every attempt will be made to administer the survey on the day on which the treatment had originally been scheduled, as symptom-related questions in the on-treatment surveys refer to the time since the preceding chemotherapy cycle. However, if this is not possible, then the survey will be administered as soon as it is practicable to do so. Participants will be contacted by email 4 months, and 1 and 2 years after treatment completion for follow-up surveys to assess employment status and related outcomes. We will call and/or send email reminders to participants asking them to take each survey, and if we do not receive a response, we will call, email, and/or send a postcard to participants to verify employment status.

All variables will be self-reported, except for cancer-related clinical variables and chemotherapy adherence. Prior research has shown limited accuracy in medically underserved breast cancer patients’ self-reporting of type of breast and axillary surgery [51]. Therefore, the research staff will review the medical records to capture clinical information about the breast cancer diagnosis and its treatment.

#### Baseline survey

Participants will complete a baseline survey that captures detailed information about demographic characteristics, place of birth, acculturation based on language (if appropriate), family structure, comorbid conditions, occupation/industry, work environment and job tenure, job tasks, union membership, and income (household and individual), which will be analyzed as a function of the federal poverty level to account for household size and secular trends [8, 52, 53]. Financial worry and distress will be assessed using the 12-item Comprehensive Score for financial Toxicity (COST). The baseline assessment will also include an assessment of symptoms using the National Cancer Institute PRO-CTCAE® and of perceived efficacy in patient-physician interactions (5-item PEPPI) [54, 55]. These measures will be combined with the measure of self-efficacy to ask an employer for accommodations (which was assessed at the time of eligibility determination). All measures used are from existing instruments that have been validated (either externally or by members of the research team) and translated into Spanish using standard methods [56].

#### On-treatment surveys

To limit participant burden, on-treatment surveys will be completed at approximately the mid-point of chemotherapy and with the last cycle only. Although the time between surveys will vary based on the regimen prescribed, the number of surveys administered to each participant will not vary. Participants whose chemotherapy regimen changes after treatment initiation will be surveyed at the projected mid-point of the regimen (on average 5–10 weeks). Such a change in adjuvant treatment regimen is rare and is not expected to affect study results. Moreover, detailed treatment information will be captured, and any effect of such a change will be evaluated in sensitivity analyses. These surveys will include an assessment of symptoms, accommodations, work status, and the two measures of self-efficacy (to ask an employer for accommodations and in patient-physician interactions).

Symptom control will be assessed using the NCI’s PRO-CTCAE®, which has been validated in English and Spanish [57, 58]. The scale may be used to measure symptoms over a period of anywhere between 1 day and 4 weeks [59]. Standard chemotherapy regimens for breast cancer have cycles varying in length from 1 to 3 weeks. Therefore, we will ask each patient to recall symptoms over the duration of the period since the last cycle of treatment. For example, if a patient is receiving chemotherapy on a 2-week cycle, her surveys will refer to her experience of symptoms over the course of the preceding 2 weeks. However, if she is receiving treatment on a 3-week cycle the questions will refer to the preceding 3 weeks.

Receipt of workplace accommodations will be assessed by asking participants about the kinds of accommodations they have requested, and whether these have been granted, including if the request was granted in full, in part, not at all, or if something else was offered in its place. Work will be measured in terms of employment status, work hours, and participant choice regarding these outcomes. Additional assessments will include self-efficacy to ask for accommodations and in patient-physician interactions.

Self-efficacy to ask for accommodations will be measured using a 5-item tool that we have developed and validated for this purpose (manuscript in preparation).

As patients in both study arms may access informational resources external to the study but which could affect the outcome, we will ask about resources they may have accessed (e.g., websites, support groups, etc.).

Financial worry and distress will be assessed using the 12-item Comprehensive Score for financial Toxicity (COST).

#### Post-treatment surveys

Post-treatment surveys will evaluate employment status, job changes, work hours, self-efficacy to ask for accommodations, disease status, ongoing or late effects of treatment, and financial stability. Financial worry and distress will be assessed using the 12-item Comprehensive Score for financial Toxicity (COST). Participants will complete follow-up surveys 4 months after completion of all active treatment (regardless of whether the last treatment is chemotherapy, radiation therapy, or surgery). Those who have completed chemotherapy and are receiving targeted therapy only (e.g., trastuzumab with or without pertuzumab) will be considered to have completed active treatment, as will those receiving endocrine therapy. Those who undergo neoadjuvant chemotherapy and are found to have residual disease at the time of surgery may undergo additional chemotherapy with capecitabine (Xeloda) or trastuzumab emtansine (T-DM1). Patients receiving capecitabine or trastuzumab emtansine in the post-operative setting will still be considered to have completed active treatment and will be eligible for the post-treatment survey 4 months after their completion of neoadjuvant chemotherapy, surgery, and radiation therapy (if indicated). Those who have undergone surgical resection but are awaiting reconstruction will also be considered to have completed active treatment. Additional long-term follow-up surveys will be administered 1 and 2 years after completion of active treatment. For all three follow-up surveys (those administered 4 months post-treatment completion as well as those administered 1 and 2 years later), the recall period for the PRO-CTCAE® symptom questions will be 4 weeks.

#### App usage

Usage tracking analytics will be provided by Curiosity Health at Cornell Tech, including whether a user accessed the app, which sections were accessed most/least, and the duration of access.

### Plans to promote participant retention and complete follow-up {18b}

Loss to follow-up is a potential problem for any longitudinal study. Our target population includes socioeconomically disadvantaged groups who may have frequent changes of address and contact information. In the BCW study [7], which targeted a similar population, we requested contact information for a friend or family member whom we could contact if we were unable to locate the participant. We also contacted participants at intermediate time points to keep them engaged. These strategies resulted in the high retention rates described in the preceding section. We will implement a similar approach in the current study.

Participants in the RCT will be given a total of $300 in CVS gift cards over the course of the 2-year study period ($50 after each survey). Participants in the usability testing and community usability testing portion of the study will receive a total of $50 in CVS gift cards upon completion of the usability testing and the Early User Experience Survey. Participants in the focus groups and interviews will receive a total of $50 in CVS gift cards for their time. Participants will be given the option to either receive physical CVS gift card(s) or electronic CVS gift card(s) as supplies allow. CVS gift cards can be used to purchase a variety of items including food and pharmacy needs, and CVS locations are conveniently located throughout New York City and New Jersey. The study team at MSK used these cards in the BCW study [7] and received positive feedback from participants regarding their ease of use.

When necessary, interview patients may be compensated up to $150 in cash to cover cost of transportation to the interview site. Throughout our recruitment efforts, the study team has noticed that many potential participants traveling from the other NYC boroughs to Manhattan have a difficult time getting to the MSK interview site. Thus, we have increased the compensation amount to support interview participation. Prior to the interview, patients will confirm if this compensation is needed and provide the amount needed.

### Data management {19}

The data collected for this study will be managed through a REDCap (Research Electronic Data Capture) database. REDCap, a data management software system supported by Memorial Sloan Kettering Cancer Center, is a tool for the creation of customized, secure data management systems including web-based data entry forms, reporting tools, and a full array of security features including user and group-based privileges with a full audit trail of data manipulation and export procedures. REDCap is maintained on MSK-owned servers that are kept in a locked server room with appropriate environmental modifications (e.g., proper ventilation, power redundancy, and fault tolerance arrangement) and backed up nightly with some back-up tapes stored off-site. The MSK Information Systems group is responsible for applying all operating system patches and security updates to the REDCap servers. All connections to REDCap utilize encrypted (SSL-based) connections. Nationally, the REDCap software is developed, enhanced, and supported through a multi-institutional consortium led by Vanderbilt University.

Any hard copy patient-information materials will be stored in locked filing cabinets in the Department of Psychiatry and Behavioral Sciences. However, all participant surveys for this study will be completed electronically and entered by the participants and/or study staff directly into REDCap. Surveys will NOT be completed through the TEAMWork app. All data will be presented in aggregate form.

During the testing phase of the mobile app, participants will interact directly with the technology, but no information they enter will be stored or transmitted, either locally on the device or centrally on the server. During the RCT, the only data that will be transmitted from users back to the study team will be usage analytics (e.g., which components of the app are accessed, how often, and for how long). No personal information will be transmitted from the app to the study team.

### Confidentiality {27}

All data files will be password-protected and stored on secure servers in the Department of Psychiatry and Behavioral Sciences at Memorial Sloan Kettering Cancer Center. Access will be restricted to personnel working directly on this project. Files that must be shared with the rest of the study team or research coordinators will be shared using MSK’s secure file transfer system. Any printed data will be stored in locked file cabinets and will be destroyed when no longer needed. Patient identifiers will be used to link information from multiple sources. We will assign patients a random number for data analysis. The matched list of patients and random study numbers will be kept by a member of the research team in a password-protected file. No attempt will be made to identify individual patients or providers beyond the use specified in this study. Publications and presentations of the data will adhere to all requirements for the protection of patient privacy and confidentiality, and no patient or provider will be individually identifiable. All data will be presented in aggregate form.

### Plans for collection, laboratory evaluation, and storage of biological specimens for genetic or molecular analysis in this trial/future use {33}

Biological specimens will not be collected.

## Statistical methods

### Statistical methods for primary and secondary outcomes {20a}

Aim 1: To refine the TEAMWork App through usability testing in English- and Spanish-speaking women who were employed pre-diagnosis and are undergoing or plan to undergo chemotherapy for breast cancer but have low confidence in requesting workplace accommodations. Focus groups will be conducted with women who are currently employed and completed chemotherapy for breast cancer treatment.

*Analysis:* The results of usability testing will be qualitatively analyzed, with responsive iterative modifications to the app implemented concurrently in English and Spanish between waves of usability testing. Each wave will include 3-4 participants per language. Focus group results will be qualitatively analyzed. Raw data obtained from the focus groups’ audio recordings and notes will be transcribed verbatim, including silences, pauses, and exclamations by the study team and the Language Services Unit of the Immigrant Health and Cancer Disparities Service at Memorial Sloan Kettering Cancer Center, following the methodology proposed by Elderkin-Thompson & Waitzkin [60]. Translation of the transcripts from Spanish to English will be done directly from the audio recordings. Once the focus groups are transcribed, data management will be performed using NVivo Pro version 12.0, which will facilitate a systematic approach for theorizing, coding, and analyzing the data. Structural coding will be used to mark responses to questions in the interview guide [61, 62]. Codes will not be predetermined by the research team (principal investigator and co-investigators), but will emerge from the data itself. Inter-rater reliability will be assessed using percent agreement and Cohen’s kappa. A codebook will be developed as a hierarchal list of themes to guide coding for major conceptual categories and then subjected to focus coding to determine minor themes and develop analytic categories. Analytic domains will be identified from the transcripts, and major and minor thematic areas will be described and used to build a theoretical outline. Through this process, hypotheses and explanatory frameworks will be generated. A similar method will be used to transcribe, code, and analyze the notes taken during the focus groups to complement the audio transcriptions. Data from the socio-demographic assessment will be entered into a password-protected Microsoft Access database and exported. Descriptive statistics will be used to describe the study participants.

Aim 2: To evaluate the effect of the TEAMWork App on job retention after completion of adjuvant therapy for breast cancer among English- and Spanish-speaking women who were employed pre-diagnosis and plan to undergo chemotherapy.

*Analysis:* To test the TEAMWork App, we have designed a 2-arm, multicenter randomized trial that is stratified by recruitment site and language (English or Spanish). The primary analysis for which this study is powered will be a direct 2-arm comparison of job retention 4 months after treatment completion. Following this, we will fit a multivariable logistic model that includes demographic and clinical variables. Per Kahan and Morris, this model will include the two stratification variables as covariates [63]. Hypothesis testing will be done using the Wald test. We will also evaluate the relationship between job retention and confidence asking for accommodations. To account for chemotherapy-specific differences, we will evaluate the chemotherapy regimen, cycle length, and number of cycles as well as symptom burden in our analyses. To identify the most relevant symptom or symptoms for inclusion in the multivariable model, we will first conduct bivariate analyses and assess the relationship between job retention and the presence of each symptom. These analyses will include symptoms reported by at least 10% of participants. This analysis will include symptoms during the entire treatment period, such that symptoms meeting the above criteria and occurring at any time during treatment will be included. As noted, randomization will be stratified by recruitment site because it is likely there are site-specific similarities that need to be balanced when making an arm-vs-arm comparison of outcomes. However, in further analyses, we will also fit mixed effects logistic regression models where the site can be viewed as a cluster. Parameter estimates from mixed models are adjusted for site-specific correlations. To evaluate long-term endpoints, we will evaluate employment status at 1 and 2 years in similar, separate analyses.

Subaim 1: To evaluate whether the effect of the app on job retention varies by characteristics, such as chemotherapy regimen, symptom burden, race/ethnicity, language, and job type.

*Analysis:* For each of the 3 logistic regressions on job retention (one for each post-treatment time-point), we will evaluate whether there is a significant interaction with the following covariates: chemotherapy regimen, symptom burden, race/ethnicity, language, and job type. Each interaction will be evaluated separately.

Aim 3: To evaluate the effect of the TEAMWork App on confidence in asking an employer for accommodations and on receipt of accommodations during and after adjuvant therapy.

*Analysis:* The relationship between the app and confidence asking for accommodations at the mid-point of chemotherapy and 4 months post-treatment will be evaluated using chi-squared tests. In subset analyses, the relationship between job retention and receipt of accommodations will be evaluated among participants who asked for accommodations.

Aim 4: To evaluate the effect of the TEAMWork App on patient efficacy in communicating with the provider and self-reported symptom burden during and following adjuvant therapy.

*Analysis:* Participants randomized to receive the app are hypothesized to have higher efficacy in communicating with the provider and lower symptom burden during and after treatment than those randomized to the informational booklet. Perceived efficacy in patient-physician interactions as measured by the PEPPI score will be compared by arm using the 2-sample *t*-test [22]. For each symptom, we will compare symptom incidence and severity by study arm. To compare symptoms, we will conduct 2 sets of analyses. One will compare the rate of symptom incidence by assigned study arm at the mid- and end-of-chemotherapy-treatment time-points separately. A second analysis will report an arm comparison of whether patients who reported a symptom at the mid-treatment survey experienced an increase, decrease, or cessation of that symptom at the end of chemotherapy treatment. The main analytic tool that will be used for these analyses is the chi-squared test.

Exploratory aim 1: To evaluate the effect of the TEAMWork App on treatment adherence.

*Analysis:* We will compare treatment adherence (RDI) by receipt of the app in a Wilcoxon rank sum test.

### Interim analyses {21b}

We have incorporated monitoring and a standalone early stopping rule to ensure the app is not causing harm. Overall, approximately 6% of BCW study [7] participants said they thought their employer had treated them unfairly. In the current study, we will call each participant who takes the 4-month survey and says her employer treated her unfairly, and we will ask about the circumstances that led to this negative outcome. Any staff making such calls will undergo sensitivity training to ensure the participant is not subject to coercion or the perception thereof as a result of the call. If there are 10 cases (per arm, at any time during the study) for whom the negative outcome is attributable to the app or booklet, we will stop the study. During the monitoring process, we will revise the intervention as needed.

### Methods for additional analyses (e.g., subgroup analyses) {20b}

All planned analyses will be performed as described above.

### Methods in analysis to handle protocol non-adherence and any statistical methods to handle missing data {20c}

As is common in longitudinal studies, we anticipate attrition in data collection. We conservatively estimate that more than 80% of study participants will complete the 4-month survey, 80% will complete the 1-year survey and 60% the 2-year survey. These estimates are based on the BCW study [7] with further detail on retention provided below. In addition to the separate analyses described above, we will also fit a mixed effects logistic regression model that uses data from all post-treatment surveys. This model will allow us to estimate 1- and 2-year employment status while using all available data. Prior to fitting this model, we will evaluate any difference between participants with complete and incomplete data since mixed models provide unbiased estimates when data are missing at random (MAR) or missing completely at random (MCAR) but not when the missingness is nonignorable (NI) [64]. Multiple imputation and pattern mixture models will be implemented following guidelines and as appropriate to the data [64].

For all main analyses, participants will be analyzed in the arm in which they were initially randomized. However, it is possible that a subset of study participants in the arm randomized to receive the app will elect not to use it. Since we are collecting detailed tracking analytics for the app, we will describe its use and, in secondary analyses, evaluate whether app arm participants who use the app are different from app arm participants who do not use it in terms of baseline characteristics and the primary endpoint.

### Plans to give access to the full protocol, participant level-data and statistical code {31c}

In accordance with the NIH Policy on Dissemination of NIH-funded Clinical Trial Information, this study is registered with ClinicalTrials.gov, and results information will be submitted no later than 1 year after the trial's primary completion date. Sharing of study data and the statistical code will be handled on a case-by-case basis.

## Oversight and monitoring

### Composition of the coordinating center and trial steering committee {5d}

Memorial Sloan Kettering Cancer Center (MSK) is the coordinating center for this study. The MSK study team will meet with the study team at each study site regularly throughout the duration of the study. In additional to the regularly scheduled (monthly) meetings, ad hoc meetings will be scheduled to address time-sensitive issues as they arise.

### Composition of the data monitoring committee, its role, and reporting structure {21a}

The Data and Safety Monitoring (DSM) Plans at Memorial Sloan Kettering were approved by the National Cancer Institute in August 2018. The plans address the new policies set forth by the NCI in the document entitled “Policy of the National Cancer Institute for Data and Safety Monitoring of Clinical Trials.”

There are several different mechanisms by which clinical studies are monitored for data safety and quality. At a departmental/PI level, there exist procedures for quality control by the research team(s). Institutional processes in place for quality assurance include protocol monitoring, compliance and data verification audits, staff education on clinical research QA, and two institutional committees that are responsible for monitoring the activities of our clinical trials programs. The committees: Data and Safety Monitoring Committee (DSMC) for Phase I and II clinical trials, and the Data and Safety Monitoring Board (DSMB) for Phase III clinical trials, report to the Deputy Physician-in-Chief of Clinical Research.

The degree of monitoring required will be determined based on the level of risk and documented.

The MSK DSMB monitors phase III trials and the DSMC monitors non-phase III trials. The DSMB/C have oversight over the following trials:

- MSK Investigator-Initiated Trials (IITs; MSK as sponsor)
- External studies where MSK is the data coordinating center
- Low-risk studies identified as requiring DSMB/C review

The DSMC will initiate review following the enrollment of the first participant or by the end of year one if no accruals and will continue for the study lifecycle until there are no participants under active therapy and the protocol has closed to accrual. The DSMB will initiate a review once the protocol is open to accrual.

### Adverse event reporting and harms {22}

Study-specific risks: Potential risks associated with this study include:

1. *Repercussions if an accommodation is requested and denied.* These repercussions may be psychological (e.g., decreased self-efficacy) or work-related (e.g. employer retaliation).
2. *Failure to report a significant symptom to the clinic team.* Participants may incorrectly believe information about symptoms they report in their survey answers will be relayed to their clinic team.
3. *Transitory distress related to thoughts about breast cancer.*
4. *Breach of confidentiality.*

Each of these specific risks is addressed below.

1. Repercussions if an accommodation is requested and denied
  *Psychological repercussions*: Participants who request accommodations at work but are denied these may find that their self-efficacy to ask for accommodations decreases rather than increases as a result. A similar outcome could occur if their attempts to improve communication with the clinic team (e.g., a request for a more potent antiemetic or for a letter to take to the employer) are not well received. All research staff will be trained to identify signs of psychological discomfort in the participants. Any patient reporting significant distress (or indicating distress through other signs) will be referred to a licensed or board-certified mental health provider for additional evaluation, support, and/or referrals as needed.
  *Work-related repercussions:* Participants who report that they have experienced negative work-related repercussions of study participation will be referred to counseling and/or pro bono legal services, as appropriate and based on participant preferences. Moreover, we have incorporated an early stopping rule into the proposal, based on this potential adverse outcome. Additional details are included in the research plan and in the Data and Safety Monitoring Plan. In brief, we will monitor participants based on their answers to a survey question about whether their employer has treated them unfairly. Overall, approximately 6% of participants in the observational Breast Cancer and the Workforce study said they thought their employer had treated them unfairly. In the current study, we will call each participant who takes the 4-month survey and says her employer treated her unfairly. In the call, we will ask about the circumstances that led to this negative outcome. If there are 10 cases (per arm, at any time during the study) for whom the negative outcome is attributable to the app or booklet, we will stop the study. During the monitoring process, we will revise the intervention as needed.
2. Failure to report a significant symptom to the clinic team
  The intervention that will be evaluated in this study includes information about common treatment-related symptoms. Additionally, patient reported symptoms will be assessed in participant surveys. The study team will not relay information about participants’ symptoms to the clinic team. Instead, participants will be expected to report any symptoms *directly* to their oncology providers. During the consent discussion participants will be informed that the information collected from them as part of the study is for research purposes only and will *not* be shared with the clinic team as part of their routine care. Furthermore, prior to initiating each survey participants will be reminded of the importance of communicating directly with the clinic team and relaying any information about symptoms directly to the appropriate physician or nurse. However, if a member of the research team or a nurse reviewer identifies an active and serious problem for a patient that should be addressed by a healthcare provider, he or she will notify the patient’s physician.
3. Transitory distress related to thoughts about breast cancer
  The risk of mild transitory distress prompted by the study intervention, control (informational brochure), usability testing, and surveys will be mitigated in several ways. First, all study materials will use clear and simple language and adhere with expert recommendations for surveys and interventions. Second, participants will be provided with contact information for the study investigators and the institutional review board at that subject’s recruitment site. All study staff who will interact with participants (i.e. in the consent process, through interviews, or when approaching participants due for surveys in clinic) will be trained in the protection of human subjects and will receive additional study-specific training to recognize and reduce participant distress. Any participant who feels distressed at any point will have the option to terminate study participation. We believe that the risk of distress due to study procedures in this in this study is low and if present, will be only mild and transitory. All research staff will be trained to identify signs of psychological discomfort in the participants. Any patient reporting significant distress (or indicating distress through other signs) will be referred to a licensed or board-certified mental health provider for additional evaluation, support, and/or referrals as needed.
4. Breach of confidentiality
  All data files will be password-protected and stored on secure servers in the Department of Psychiatry and Behavioral Sciences at Memorial Sloan Kettering Cancer Center. Access will be restricted to personnel working directly on this project. Files that must be shared with the rest of the study team or research coordinators will be shared using MSK’s secure file transfer system. Any printed data will be stored in a locked file cabinet and will be destroyed when no longer needed. Patient identifiers will be used to link information from multiple sources. We will assign patients a random number for data analysis. The matched list of patients and random study numbers will be kept by a member of the research team in a password-protected file. No attempt will be made to identify individual patients or providers beyond the use specified in this study. Publications and presentations of the data will adhere to all requirements for the protection of patient privacy and confidentiality, and no patient or provider will be individually identifiable. All data will be presented in aggregate form.

### Frequency and plans for auditing trial conduct {23}

Reports will be generated to monitor participant accrual and completeness of registration data. Routine data quality reports will be generated to assess missing data and inconsistencies every month. Accrual rates and extent and accuracy of evaluations and follow-up will be monitored periodically throughout the study period and potential problems will be brought to the attention of the study team for discussion and action. Random-sample data quality and protocol compliance audits will be conducted by the study team, at a minimum of twice per year, or more frequently if indicated.

### Plans for communicating important protocol amendments to relevant parties (e.g., trial participants, ethical committees) {25}

Each change to the protocol document must be organized and documented by MSK and approved first by the MSK IRB/PB. Protocol amendments that affect MSK only (e.g., change in MSK Co-Investigator, MSK translation, etc.) do not require IRB review at the participating sites. All other protocol amendments will be immediately distributed to each participating site upon receipt of MSK IRB/PB approval.

Each participating site must obtain IRB approval for all amendments *within 90 calendar days* of receipt of the amended MSK IRB/PB documents. If the amendment is the result of a safety issue or makes eligibility criteria more restrictive, participating sites will not be permitted to continue to enroll new participants until site IRB approval of the revised protocol documents is granted and submitted to MSK.

Participating sites must notify MSK research staff of any site-initiated amendments/modifications. Each participating site must provide all site IRB approvals for amendments/modifications and the most current approved version of the site informed consent form and HIPAA authorization at the time of approval. Documents must be submitted to MSK on a continuing basis.

### Dissemination plans {31a}

The study PI will work to disseminate the findings through her network of clinicians, researchers, and professional and patient organizations, including the Alliance in Clinical Trials Oncology, the American Society of Clinical Oncology, and other oncology associations in which she participates, as well as Cancer and Careers and LatinaSHARE, two nonprofit organizations with whom she collaborates and has close professional ties. The PI will also disseminate her research through journal publications and conference presentations to reach clinicians, social workers, and administrators who can, in turn, raise awareness with patients. She will publish her findings throughout the study so that others may benefit from their findings at each research stage. If shown to be effective, TEAMWork can be implemented in cancer clinics nationally and modified to help patients with other chronic diseases. The research team will work to make the app publicly available for smartphone users.

## Discussion

The COVID-19 pandemic began in the last quarter of year 2 of the grant, and New York City soon became its epicenter. As a result of the pandemic, study procedures were temporarily halted, as institutional personnel were redeployed to work in clinical roles. Additionally, on-site participant recruitment and interviewing were halted at MSK as well as Montefiore and Lincoln Hospital, as per institutional mandates. Over the course of the following months, we were able to adjust our procedures at all study sites to accommodate remote recruitment, consenting, and interviewing. To maximize social distancing, the final vetting the app content in Spanish was modified to an interview format, which participants preferred to virtual focus groups. Importantly, the public health emergency which justified our use of remote recruitment and consenting procedures has also served as a model for how verbal consent has been used appropriately for minimal-risk studies such as this one. Moving forward, we will continue to recruit and consent study participants remotely, which will allow us to reach greater numbers of potential participants during the eligibility window (between their diagnosis and initiation of chemotherapy).

The pandemic affected not only our ability to recruit study participants as described above, but also the delivery of the final study app. In March 2020, the original app developer was pulled away to a COVID-related project, and for several months our project was put on hold at their company, with delivery of the major final app modifications being repeatedly postponed by the developer. Later in the year, we were advised that the company had dissolved without having delivered the final product. Fortunately, we were able to mobilize institutional resources at MSK and identify a suitable contractor to take over the project, although there were additional delays in obtaining the app code that had been created by the first developer. Since early 2021, we have been working with this MSK contractor and with the Technology Division at MSK, and we now have a completed and fully functional app.

Finally, the TEAMWork app includes a set of videos that model communication with an employer, coworkers, and the clinic team. The video scripts were vetted by professional stakeholders and patients, and filming was to begin using the MSK Communication Skills lab in early 2020. Due to COVID-19, the lab was closed, and it was no longer possible to film the videos on site. Instead, we filmed the 54 videos (27 per language) using online technology and worked with the MSK Video Department to ensure that video quality was optimized.

## Trial status

The randomized clinical trial is open to accrual at all study sites. The most recent amendment (version 19) was approved on February 16, 2022. Study recruitment began (for usability testing) on August 30, 2019. It is anticipated that recruitment in the RCT will continue through the end of 2023.

## Supplementary Information

**Additional file 1. **SPIRIT Checklist for *Trials*.

## Data Availability

There are no existing contractual agreements that limit access to the data for investigators.

## Abbreviations

BCW: Breast Cancer and the Workforce [7]
COST: Comprehensive Score for financial Toxicity
IRB: Institutional Review Board
MSK: Memorial Sloan Kettering Cancer Center
NYC: New York City
PEPPI: Perceived Efficacy in Patient-Physician Interactions
PRO-CTCAE®: Patient-Reported Outcomes version of the Common Terminology Criteria for Adverse Events
RCT: Randomized controlled trial
REDCap: Research Electronic Data Capture
T-DM1: Trastuzumab emtansine
TEAMWork: Talking to Employers And Medical staff about Work
